# Characterizing the Relationship Between Arterial Carbon Dioxide Trajectory and Serial Brain Biomarkers with Central Nervous System Injury During Veno-Venous Extracorporeal Membrane Oxygenation: A Prospective Cohort Study

**DOI:** 10.1007/s12028-023-01923-x

**Published:** 2024-02-01

**Authors:** Sonny Thiara, Sophie Stukas, Ryan Hoiland, Cheryl Wellington, Mike Tymko, George Isac, Gordon Finlayson, Hussein Kanji, Kali Romano, Veronica Hirsch-Reinshagen, Mypinder Sekhon, Donald Griesdale

**Affiliations:** 1 Division of Critical Care Medicine, Department of Medicine, Faculty of Medicine, Vancouver General Hospital, University of British Columbia, Room 2438, Jim Pattison Pavilion, 2nd Floor 855 West 12th Avenue, Vancouver, BC V5Z 1M9 Canada; 2 Department of Pathology and Laboratory Medicine, University of British Columbia, Vancouver, BC Canada; 3 Department of Anesthesiology, Pharmacology and Therapeutics, Faculty of Medicine, Vancouver General Hospital, University of British Columbia, Vancouver, BC Canada; 4 Department of Emergency Medicine, Faculty of Medicine, Vancouver General Hospital, University of British Columbia, Vancouver, BC Canada

**Keywords:** Veno-venous extracorporeal membrane oxygenation, Brain biomarkers, Central nervous system

## Abstract

**Background:**

Central nervous system (CNS) injury following initiation of veno-venous extracorporeal membrane oxygenation (VV-ECMO) is common. An acute decrease in partial pressure of arterial carbon dioxide (PaCO_2_) following VV-ECMO initiation has been suggested as an etiological factor, but the challenges of diagnosing CNS injuries has made discerning a relationship between PaCO_2_ and CNS injury difficult.

**Methods:**

We conducted a prospective cohort study of adult patients undergoing VV-ECMO for acute respiratory failure. Arterial blood gas measurements were obtained prior to initiation of VV-ECMO, and at every 2–4 h for the first 24 h. Neuroimaging was conducted within the first 7–14 days in patients who were suspected of having neurological injury or unable to be examined because of sedation. We collected blood biospecimens to measure brain biomarkers [neurofilament light (NF-L); glial fibrillary acidic protein (GFAP); and phosphorylated-tau 181] in the first 7 days following initiation of VV-ECMO. We assessed the relationship between both PaCO_2_ over the first 24 h and brain biomarkers with CNS injury using mixed methods linear regression. Finally, we explored the effects of absolute change of PaCO_2_ on serum levels of neurological biomarkers by separate mixed methods linear regression for each biomarker using three PaCO_2_ exposures hypothesized to result in CNS injury.

**Results:**

In our cohort, 12 of 59 (20%) patients had overt CNS injury identified on head computed tomography. The PaCO_2_ decrease with VV-ECMO initiation was steeper in patients who developed a CNS injury (− 0.32%, 95% confidence interval − 0.25 to − 0.39) compared with those without (− 0.18%, 95% confidence interval − 0.14 to − 0.21, *P* interaction < 0.001). The mean concentration of NF-L increased over time and was higher in those with a CNS injury (464 [739]) compared with those without (127 [257]; *P* = 0.001). GFAP was higher in those with a CNS injury (4278 [11,653] pg/ml) compared with those without (116 [108] pg/ml; *P* < 0.001). The mean NF-L, GFAP, and tau over time in patients stratified by the three thresholds of absolute change of PaCO_2_ showed no differences and had no significant interaction for time.

**Conclusions:**

Although rapid decreases in PaCO_2_ following initiation of VV-ECMO were slightly greater in patients who had CNS injuries versus those without, data overlap and absence of relationships between PaCO_2_ and brain biomarkers suggests other pathophysiologic variables are likely at play.

**Supplementary Information:**

The online version contains supplementary material available at 10.1007/s12028-023-01923-x.

## Introduction

Veno-venous extracorporeal membrane oxygenation (VV-ECMO) enables ex vivo gas exchange (oxygenation and removal of carbon dioxide) in critically ill patients with acute respiratory failure [1] and mitigates ventilator-induced lung injury [1, 2]. The use of VV-ECMO to treat patients with refractory acute respiratory distress syndrome has increased in recent years because of improvements in portability [3], simplicity of the extracorporeal circuit [4], and the emergence of the coronavirus disease 2019 (COVID-19) pandemic [5, 6].

Although VV-ECMO can be lifesaving, its use is associated with significant complications [7]. Specifically, central nervous system (CNS) injury (e.g., intracerebral hemorrhage and/or ischemic infarction) following the initiation of VV-ECMO is associated with increased mortality and adverse long-term functional outcomes [8–11]. Historical cohort studies suggest that among adult patients with acute respiratory failure undergoing VV-ECMO, the incidence of CNS injury ranges from 7 to 50% [9]. Importantly, in-hospital mortality in patients with CNS injury is greater than 75%, compared with less than 40% in those without CNS injury [8, 9].

One potential mechanism of CNS injury following VV-ECMO initiation is thought to be related to a precipitous decrease in partial pressure of arterial carbon dioxide (PaCO_2_) and consequent cerebral vasoconstriction and hypoperfusion [12, 13], which would also worsen CNS injury, especially ischemic, resulting from another mechanism. Previous studies have linked PaCO_2_ reductions following ECMO to heterogeneous composite definitions of CNS injuries [14, 15] encompassing seizures, ischemic stroke, intracranial hemorrhage, or brain death [9]. These studies have also modeled various “exposures” of PaCO_2,_ such as the immediate reduction on initiation of VV-ECMO [14], and comparing pre-VV-ECMO value with the PaCO_2_ at 24 h following initiation [15]. However, ascribing fixed thresholds of PaCO_2_ does not adequately reflect the trajectory of this continuous variable over time. In addition, the timing and characterization of CNS injury in patients undergoing VV-ECMO are also unclear. The diagnosis of CNS injury relies on clinical examination or head computed tomography (CT) [16], both of which are limited in patients undergoing VV-ECMO because of patient sedation and transport logistics, respectively. Moreover, clinical examination may underestimate the true occurrence of CNS injury [17]. Thus, alternate methods to identify patients with a CNS injury in a timely matter are needed.

Blood-based brain injury biomarkers have been increasingly studied to characterize the timing, severity, and mechanism(s) of CNS injury in traumatic brain injury and Alzheimer’s disease [18–21]. Glial fibrillary acidic protein (GFAP) is a component of the astrocytic cytoskeleton, highly specific to the CNS, and reflects astroglia activation and/or injury [18]. Phosphorylated-tau protein (p-tau 181) and neurofilament light (NF-L) reflect injury primarily in axons [19–21] and myelinated white matter. Each of these biomarkers have been used to assess the severity of ischemic brain injury [22], and they are instantaneously released during brain hypoxia [23], thereby holding promise as acute diagnostic tools to assess of CNS injuries in critically ill patients, despite the current evidence for their use being mostly limited to prognostication.

**Fig. 1:**
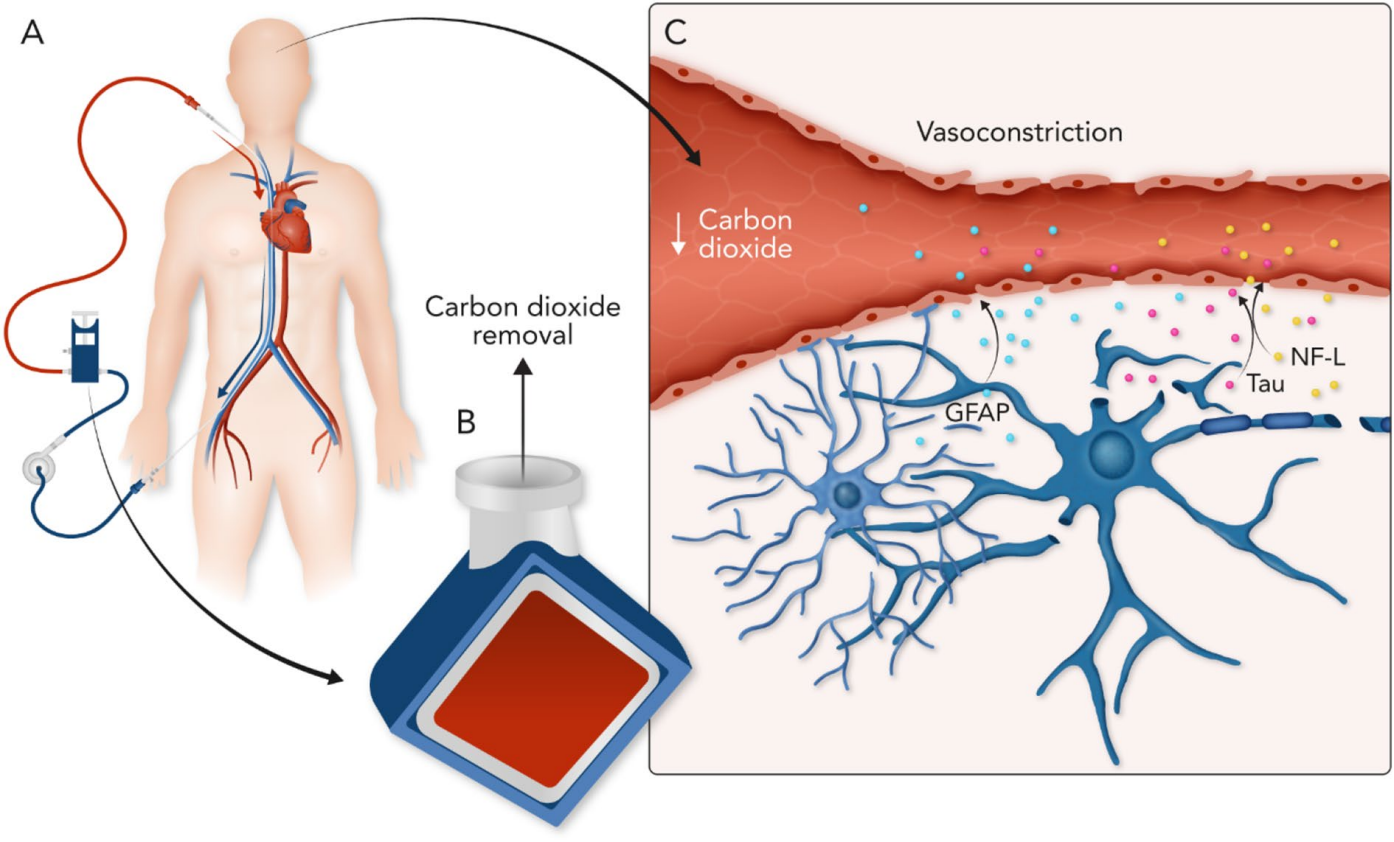
Proposed paradigm for neurological injury on VV-ECMO. During VV-ECMO, venous blood is removed and circulates through a membrane where oxygen is added, and CO_2_ is removed (**a** and **b**). The low CO_2_ within the cerebral vasculature results in vasoconstriction and decreased cerebral blood flow (**c**), and the resultant neuronal injury leads to release of neurological biomarkers (GFAP, tau, and NF-L) in the bloodstream. *GFAP* glial fibrillary acidic protein, *NF-L* neurofilament light, *VV-ECMO* veno-venous extracorporeal membrane oxygenation

Therefore, we conducted a prospective observation cohort study in patients undergoing VV-ECMO with three aims. First, we sought to characterize the relationship between acute reductions in PaCO_2_ following initiation of VV-ECMO and CNS injury detected with clinical examination. We hypothesized that more rapid reduction in PaCO_2_ around the time of initiation of VV-ECMO would be associated with CNS injury. Second, we sought to assess the association between biomarkers of neurologic injury and CNS injuries detected with clinical examination. We hypothesized that serum levels would be significantly greater in patients with CNS injuries compared with those without. Third as an exploratory analysis, we aimed to examine the relationship between changes in PaCO_2_ following the initiation of VV-ECMO and biomarkers of brain injury. We hypothesized that greater reductions in PaCO_2_ following initiation of VV-ECMO would be associated with increased serum biomarker levels (Fig. 1).

## Methods

We conducted a prospective observational cohort study of 59 consecutive adult patients who received VV-ECMO at Vancouver General Hospital intensive care unit (ICU). University of British Columbia Clinical Research Ethics Board approval was obtained for the study (H21-00033/H20-00971), and informed consent was obtained from the patient’s legal authorized representative.

### Study Setting, Management, and Population

The ICU at Vancouver General Hospital is a closed, 40 bed, mixed medical-surgical unit with a 1:1 nursing-to-patient ratio and intensivists with subspecialty training in VV-ECMO. On-site perfusion specialists are in attendance to guide ongoing provision of VV-ECMO. Cannulation with percutaneous Seldinger technique is guided by ultrasound [24], with standardized right femoral vein access (23–27 Fr) and right internal jugular vein (15–19 Fr) return lines. Cardiohelp (Gothenburg, Sweden) or Sorin (London, UK) devices are used. Unfractionated heparin (5,000 unit intravenous bolus) is administered prior to initiation of VV-ECMO, and the sweep gas is set between 1 and 3 L/min to minimize precipitous reductions in PaCO_2_. Heparin infusions are titrated using a standardized protocol for a partial thromboplastin time target of 50–70 s while on VV-ECMO. Other management decisions are standardized including mean arterial pressure > 65 mm Hg, arterial oxygen tension (60–100 mm Hg), normothermia (36–37.5 °C). The primary sedatives used are intravenous propofol, hydromorphone, and ketamine. The primary vasopressor used is norepinephrine. Management is reflective of Extracorporeal Life Support Organization (ELSO) recommendations [24].

We included patients who were older than 18 years of age undergoing VV-ECMO for acute respiratory failure. We excluded patients with either a preexisting history of chronic CNS injury (or neurodegenerative disorder) or preexisting known CNS injury (traumatic brain injury, stroke, intracranial hemorrhage, hypoxic ischemic brain injury following cardiac arrest).

### Data Sources, Measurement, and Outcomes

In addition to demographic data, we collected the following using a Research Electronic Data Capture (H14-00930) database [23], based on ELSO definitions: VV-ECMO related complications (e.g., CNS injury, bleeding, organ failure, infection, death), circuit complications (e.g., oxygenator failure, air embolism, pump thrombosis), and clinical physiological parameters (e.g., hourly mean arterial pressure, body temperature, sedative and vasopressor doses). Timing of arterial blood gas measurements were obtained prior to initiation of VV-ECMO and at every 2–4 h for the first 24 h. Biospecimens collected for the analysis of brain biomarkers were obtained at four time points via an in situ arterial line: immediately prior to VV-ECMO initiation, and at 1 h, 24 h, and 7 days following the initiation of VV-ECMO. Samples were collected in serum separator tubes (Becton & Dickinson, Vacutainer, 367986), set upright in the dark for 10 min and then centrifuged at 600*g* for 10 min, with the serum supernatant aliquoted into cryovials and immediately frozen in a − 80 °C freezer. Plasma concentrations of NF-L and GFAP were quantified using the Neuro-4-plex-E advantage assay (cat no. 103670) and p-tau-181 V2 advantage assay (cat no. 103714) using the Quanterix Simoa HD-X platform following the manufacture’s protocol.

### Neurologic Outcomes

CNS injury was defined as either a new intracranial hemorrhage or infarct on CT imaging of the brain. Neuroimaging is conducted as part of routine care within the first 7–14 days for most patients managed with VV-ECMO in our institution who require ongoing intravenous sedation, thereby confounding the clinical examination as determined by clinician judgment. Patients without neurological deficits off sedation and who did not have a CT scan were considered to not have CNS injury.

### Statistical Analysis

#### Relationship Between PaCO_2_ and Neurologic Outcome

We first visually assessed the relationship by plotting PaCO_2_ (connected line plot for each patient) over time stratified by injury status. Because of the nonlinear relationship between PaCO_2_ and time, we performed a logarithmic transformation of PaCO_2_ prior to fitting our model. We then performed a mixed-effects linear regression of ln(PaCO_2_) on injury (dichotomous variable), specifying “patient” as a random effect (Stata command *xtreg*). In order to assess for effect measure modification, we also included an interaction variable of time and injury.

To assess previously published relationships of changes in PaCO_2_ and CNS injury [14, 15], we dichotomized PaCO_2_ exposure in three separate ways. First, we compared those patients with an absolute change of PaCO_2_ (∆PaCO_2_) of ≥ 27 mm Hg compared with those with a ∆PaCO_2_ < 27 mm Hg on initiation of VV-ECMO (threshold identified by Luyt et al. [14]). Second, we calculated the pre-post percentage (PP%) as the difference between the PaCO_2_ obtained immediately prior to VV-ECMO and 24-h after initiation, divided by the pre-VV-ECMO PaCO_2_ at the 50% threshold (per Cavayas et al. [15]). Third, we calculated the maximum-minimum percentage (MM%) as the highest minus the lowest PaCO_2_ in the first 24 h of VV-ECMO divided by the pre-VV-ECMO PaCO_2_ (a novel metric). We then assessed the relationship between all three PaCO_2_ variables with CNS injury using univariable logistic regression. Finally, as part of a post hoc sensitivity analysis, we explored different thresholds for ΔPaCO_2_ MM%.

#### Relationship Between Brain Biomarkers and CNS Injury

We first visually assessed NF-L, GFAP and *p*-tau 181 over time (connected line plot for each patient) and stratified by injury status. Because of the nonlinear relationship between NF-L and GFAP with time, we performed a logarithmic transformation of the biomarker values prior to fitting a model. We then performed separate mixed-effects linear regression for each biomarker using CNS injury as a dichotomous predictor variable and specifying “patient” as a random effect (Stata command *xtreg*). To assess for effect measure modification, we also included an interaction variable of time (indicator variables) and injury.

### Post Hoc Exploratory Analysis

To explore the effects of ΔPaCO_2_ on serum levels of neurological biomarkers, we performed separate mixed methods linear regression for each biomarker using three PaCO_2_ exposures (reduction on initiation of VV-ECMO ≥ 27 mm Hg, ΔPaCO_2_ PP% > 50% and ΔPaCO_2_ MM% ≥ 50%) as a dichotomous predictor variable and specifying “patient” as a random effect in patients.

All analyses were two-sided, we considered a* P* value < 0.05 statistically significant, and analyses were performed with Stata 16.0 (StataCorp. 2019. Stata Statistical Software: Release 16. College Station, TX: StataCorp LLC).

## Results

We enrolled 59 patients between April 1st, 2020, and November 30th, 2021. The mean (standard deviation) age was 50 (10) years, and 11 (17%) patients were female. Fifty (85%) patients required VV-ECMO for respiratory failure secondary to COVID-19 (Table 1). Twelve patients (20%) developed a CNS injury post VV-ECMO initiation, of whom nine patients had an intracranial hemorrhage and three patients had an ischemic infarct. Median time to diagnosis was 9.5 (7–15.5) and 17.5 (3–47.5) days from the initiation of VV-ECMO for patients with an intracranial hemorrhage or ischemic stroke, respectively. Overall survival to ICU discharge 66% (39/59), which was 25% (3/12) in the CNS injury group and 77% (36/47) in those without CNS injury. All patients had one arterial blood gas (ABG) prior to VV-ECMO, and patients without CNS injury had a mean (standard deviation) of 8 (2) ABG measurements in the first 24 h whereas patients with CNS injury had 9 (2). Median PaCO_2_ pre VV-ECMO was 68 mm Hg (54–76) in the CNS injury group and 70 mm Hg (58–94) in those without CNS injury (odds ratio [OR] 1.02, 95% confidence interval [CI] 0.99 to 1.04).

**Table 1:** Characteristics of patients receiving VV-ECMO included in the cohort stratified by the absence or presence of CNS injury

Characteristics	No CNS injury (*n* = 47)	CNS injury (*n* = 12)
Age, years, mean (SD)	52 (10)	50 (12)
Female sex, *n* (%)	8 (17)	3 (25)
Indication for VV-ECMO, *n* (%)		
COVID-19	40 (85)	10 (83)
ILD	1 (2)	1 (8)
Post lung transplant	0	1 (8)
ARDS bacterial PNA	1 (2)	0 (0)
ARDS trauma/inhalation/drug	5 (11)	0 (0)
Femoral-Jugular cannulation strategy, *n* (%)	40 (87)	10 (83)
Neuroimaging, *n* (%)	33 (70)	12 (100)
Time to neuroimaging, days, median (IQR)	8 (1–11)	8 (3–13)
Days of mechanical ventilation pre ECMO, median (IQR)	4 (3–8)	5 (3–9)
VV-ECMO duration, days, median (IQR)	22 (8–30)	14 (9–22)
INR, mean (SD)	1.2 (0.2)	1.1 (0.2)
PTT, seconds, mean (SD)	41 (41)	62 (48)
Platelet count × 10^9^/L, mean (SD)	232 (111)	184 (137)
PF pre-VV-ECMO, mean (SD)	74 (20)	66 (18)
PaCO_2_ pre VV-ECMO, mm Hg, median (IQR)	67 (54–76)	71 (63–96)
PaCO_2_ post VV-ECMO start, mm Hg, median (IQR)	60 (46–84)	67 (61–80)
PaCO_2_ 24-h post VV-ECMO start, mm Hg, median (IQR)	48 (43–56)	50 (45–52)
PaCO_2_ reduction on initiation of VV-ECMO ≥ 27 mm Hg, *n* (%)	22 (47)	4 (33)
∆PaCO_2 ≥_ 50_%pre-post%_, *n* (%)^a^	4 (8)	3 (25)
∆PaCO_2 ≥_ 50%_max–min%_, *n* (%)^b^	12 (26)	9 (75)
∆PaCO_2_%_max–min%_, median (IQR)^b^	39 (28–58)	62.5 (47–71.5)

*ARDS* acute respiratory distress syndrome, *CNS* central nervous system, *COVID-19* coronavirus disease of 2019, *ILD* interstitial lung disease, *INR* international normalized ratio, *IQR* interquartile range, *max* maximum, *min* minimum, *MV* mechanical ventilation, *PaCO*_*2*_ partial pressure of arterial carbon dioxide, *SD* standard deviation, *PF* arterial oxygen partial pressure:fraction of inspired oxygen, *PNA* pneumonia, *PTT* partial thromboplastin time, *VV-ECMO* veno-venous extracorporeal membrane oxygenation

^a^24-h post-ECMO PaCO_2_-pre-ECMO PaCO_2_)/pre-ECMO PaCO_2_

^b^CO_2_ max first 24 h-CO_2_ min first 24 h/Pre-ECMO PaCO_2_

### Relationship Between PaCO_2_ and Neurologic Outcome

**Fig. 2:**
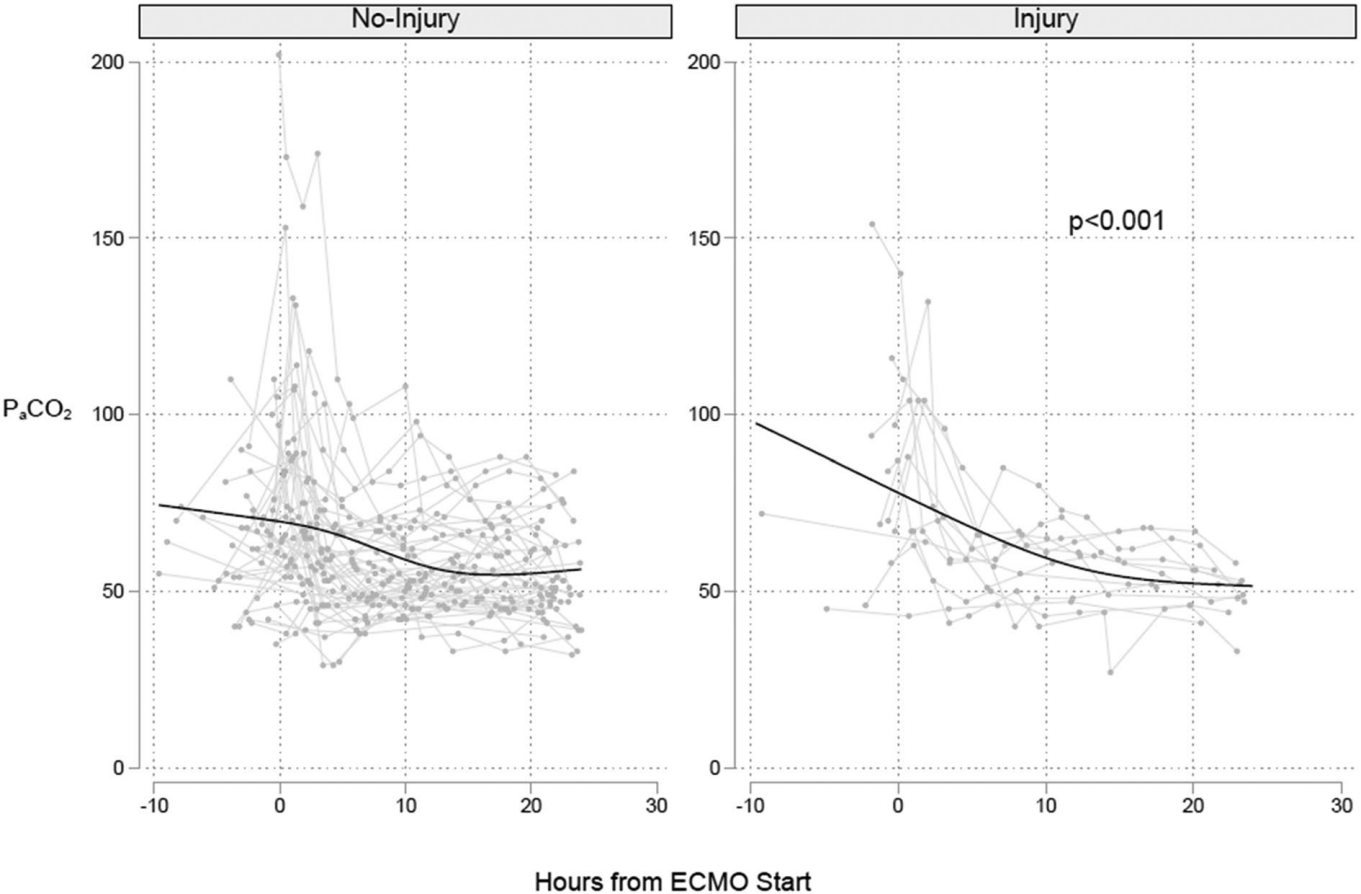
Arterial carbon dioxide trajectories on VV-ECMO in patients with and without subsequent CNS injury. Connected line plots of PaCO_2_ (*y*-axis) versus time (*x*-axis) in patients without (left panel) and with (right panel) a CNS injury. Each light gray line is an individual patient. The black line is a predicted curve generated using a restricted cubic splines model. CNS, central nervous system, PaCO_2_, partial pressure of arterial carbon dioxide, VV-ECMO, veno-venous extracorporeal membrane oxygenation

PaCO_2_ values over the first 24 h were analyzed over time in patients with and without CNS injury (Fig. 2). After logarithmic transformation, PaCO_2_ decreased over time in all patients (− 0.21% per 10 min, 95% CI − 0.17 to − 0.24). There was effect measure modification of the PaCO_2_ over time by CNS injury (*P* interaction < 0.001). Patients with CNS injury had a steeper reduction in PaCO_2_ by − 0.32% (95% CI − 0.25 to − 0.39) for each 10 min compared to a reduction of PaCO_2_ by − 0.18% (95% CI − 0.14 to − 0.21) in those without CNS injury. Post hoc analysis of various PaCO_2_ thresholds is shown in the Electronic Supplementary Material (E-Table 1). Accordingly, ΔpaCO_2_ MM% ≥ 50% in first 24 h was associated with an increased odds of CNS injury (OR 8.8, 95% CI 2.0–37.8). However, neither PaCO_2_ reduction on initiation of VV-ECMO ≥ 27 mm Hg (OR 1.0, 95% CI 0.9–1.0), nor ΔPaCO_2_ PP% > 50% (OR 3.6, 95% CI 0.7–18.9) were associated with CNS injury.

### Relationship Between Brain Biomarkers and CNS Injury

**Fig. 3:**
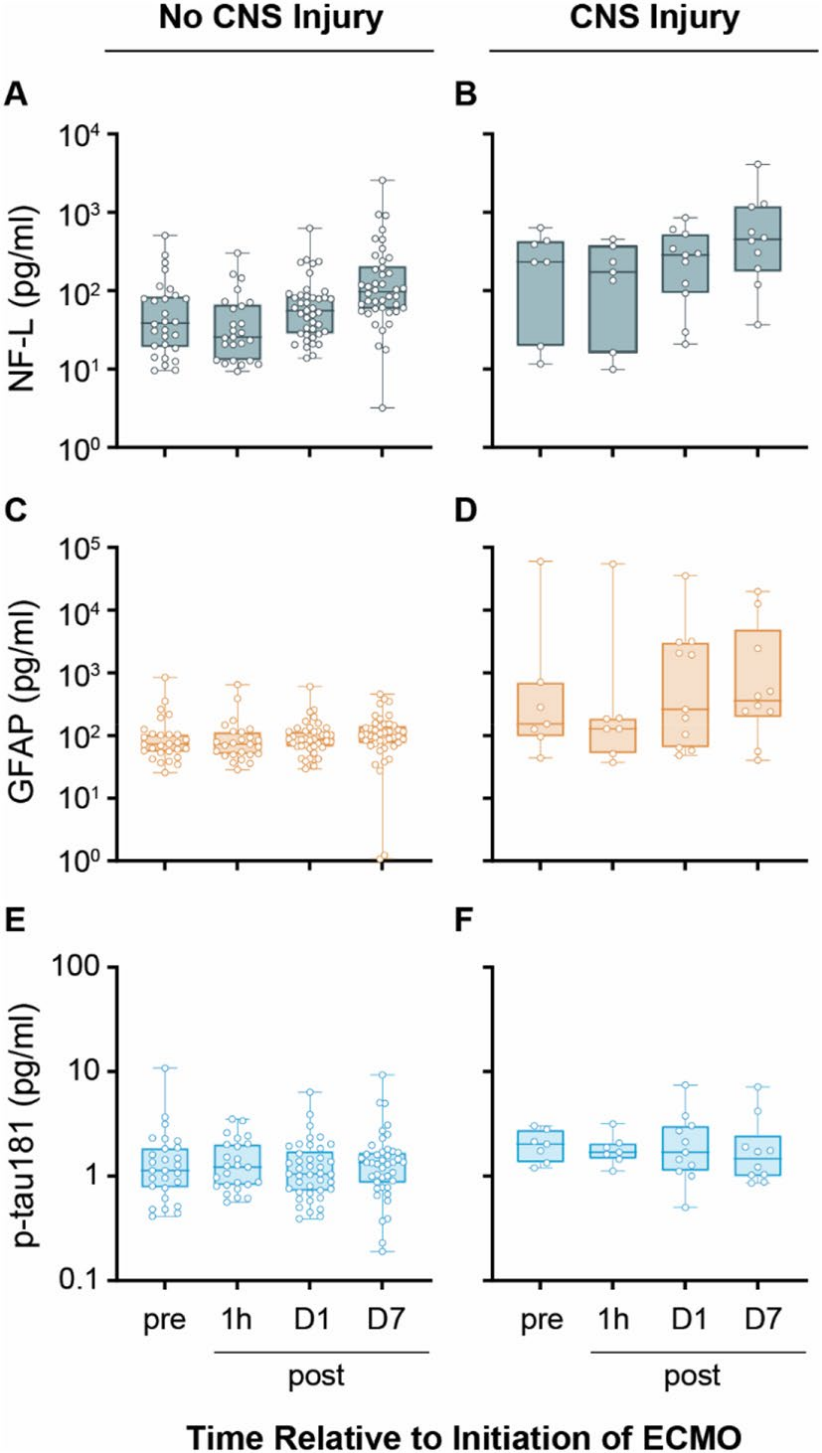
Neurological biomarker trajectories in the first week following initiation of VV-ECMO in patients with and without subsequent CNS injury. NF-L (**a**, **b**), GFAP (**c**, **d**), and p-tau 181 (**e**, **f**) were quantified in samples taken in the 24-h prior to, and 1 h, 1 day, and 7 days post initiation of VV-ECMO. Patients were stratified based on the subsequent absence [*n* = 47, no CNS injury graphs (**a**, **c**, **e**)] or presence [*n* = 12, CNS injury graphs (**b**, **d**, **f**)] of CNS injury. Box plots show median values and highlight interquartile range with maximum and minimum ranges shown. *CNS* central nervous system, *GFAP* glial fibrillary acidic protein, *NF-L* neurofilament light, *p-tau 181* phosphorylated-tau 181, *VV-ECMO* veno-venous extracorporeal membrane oxygenation

Baseline brain biomarker levels stratified by CNS injury are presented in E-Table 2 and Fig. 3. The mean (standard deviation) change in NF-L level from baseline to day 7 was higher in the CNS injury group (464 [739] pg/ml) compared to those without (127 [257] pg/ml) (*P* < 0.001). NF-L levels were higher at each time point (including pre VV-ECMO) in the CNS injury group and increased over time for both groups. There was no interaction of time by CNS injury for NF-L (*P* interaction = 0.43). For GFAP, the mean change in level from baseline to day 7 was higher in the CNS injury group (4,278 [11,653] pg/ml) compared to those without (116 [108] pg/ml) (*P* < 0.001). GFAP did not increase over time and there was no interaction between time and CNS injury (*P* interaction = 0.23). There was no difference in p-tau 181 change over time in the CNS injury group compared to those without, (2.1 [1.6] vs. 1.5 [1.4] pg/ml, *P* = 0.14), and there was no interaction between injury and time (*P* interaction = 0.63).

### Post Hoc Exploratory Analysis of Changes in PaCO_2_ and Biomarkers of Neurologic Injury

E-Fig. 1 is the mean NF-L, GFAP and tau over time in patients stratified by the three thresholds of ΔpaCO_2_ (≥ 27 mm Hg vs. < 27 mm Hg; PP% ≥ 50% vs. < 50%; MM% ≥ 50% vs. < 50%). There were no differences in mean NF-L, GFAP or p-tau 181 at baseline and no significant interaction for time for NF-L, GFAP or tau.

## Discussion

We present a prospective observational study investigating the role of PaCO_2_ reduction in VV-ECMO associated CNS injury pathophysiology and shed light on the relationships of brain biomarkers to CNS injury in VV-ECMO. Although we observed a greater statistical reduction in PaCO_2_ in those with a CNS injury compared with those without, visual examination of this relationship does not support an overwhelming difference in the magnitude of this relationship between groups. Previously defined reductions of PaCO_2_ on initiation of VV-ECMO and the comparison of values 24 h apart, are likely not the sole explanation for the etiology of VV-ECMO associated CNS injury and a MM% of PaCO_2_ in first 24 h of VV-ECMO may be better exposure variable to represent the PaCO_2_ effects on CNS injury. In the biomarker analysis, we demonstrated that systemically circulating levels of NF-L and GFAP were greater in patients who developed a CNS injury. Furthermore, there was a progressive increase in NF-L during the first 7 days regardless injury status. Finally, we did not observe relationships between various thresholds of PaCO_2_ reduction following initiation of VV-ECMO and systemically circulating brain biomarker levels.

Our study focused on evaluating the proposed pathophysiology relating the acute reduction of PaCO_2_ to CNS injuries. Although we observed a slightly steeper reduction in PaCO_2_ in patients with CNS injury compared with those without, given the significant overlap in data, similarity in the appearance of the curves and small number of outcomes (*n* = 12), it is questionable that PaCO_2_ is the sole driver of the pathophysiology for VV-ECMO associated CNS injury. Instead, our data point to a more complex picture in which alternative and perhaps unknown mechanisms, are at play. Luyt et al. examined 135 consecutive patients on VV-ECMO and demonstrated that intracranial bleeding was associated with ∆PaCO_2_ decrease ≥ 27 mm Hg (OR 6.0, 95% CI 1.2–30.0) following initiation of VV-ECMO [14]. In a large historical analysis of 11,972 patients in the Extracorporeal Life Support Organization Registry, Cavayas et al. showed that a ΔPaCO_2_ PP% reduction > 50% was associated with neurological complications (OR 1.7, 95% CI 1.3–2.3) [15]. In each case, the definition of CNS injuries has been heterogeneous with a composite outcome of cerebral hemorrhage, ischemia, seizures, and neurological brain death [15]. Further, retrospective designs hinder the strength of these conclusions. Clinical assessment of CNS injury may also underestimate pathology determined CNS injury. Given our hypothesis that the trajectory of PaCO_2_ changes upon VV-ECMO initiation may lead to CNS injury, we explored various thresholds of ΔPaCO_2_ MM% in first 24 h and found that a MM% ≥ 50 (OR 8.8, 95% CI 2.0–37.8) may represent a PaCO_2_ exposure that requires further validation in its effects on CNS injury. CNS injury is likely multifactorial in VV-ECMO and other mechanisms unrelated to PaCO_2_ likely contribute, a recent cohort study identified that lower PaO_2_ 24 h post cannulation is associated with the development of CNS injury [25].

Although these studies point to PaCO_2_ and PaO_2_ changes resulting in CNS injury, there are major limitations to these studies. The studies by Akbar and Cavayas were retrospective studies that used ABG values that were 24 h apart to define the change in PaO_2_ and PaCO_2_ that resulted in injury [15, 25]. In examining our multiple daily ABG data, we can see the trajectory of PaCO_2_ can be drastically different in the intervening period when two measurements are taken 24 h apart and these results may reflect a statistical phenomenon related to large sample sizes in ELSO registry cohorts. The study by Luyt was also retrospective and highlights that no prospective study has reproduced a previously identified PaCO_2_ exposure resulting in CNS injury, with all exposures being defined in retrospect based on available data [14].

We also observed that two biomarkers of neurologic injury, NF-L and GFAP, were elevated in patients who developed a CNS injury. Furthermore, NF-L was elevated prior to initiation of VV-ECMO in those patients who developed CNS injury. This latter finding suggests that there could be an unrecognized neurologic injury prior to cannulation that is related subsequent determination of CNS injury post cannulation via clinical exam or neuroimaging. Unfortunately, current diagnostic modalities are limited in this patient population. For example, clinical examination is often confounded by sedative use, and CT imaging is challenging in patients who are physiologically unstable [16]. Therefore, brain biomarkers represent an objective and quantitative diagnostic tool that might overcome these limitations and identify at-risk patients who may develop CNS injury following VV-ECMO initiation. Given that brain biomarkers are associated with adverse outcome in patients with neurological injury after cardiac arrest and traumatic brain injury, there is promise for their use in patients requiring VV-ECMO [22, 26]. Further, Hoiland et al. demonstrated release of NF-L and GFAP in patients with brain tissue hypoxia, a physiologic perturbation which may occur following VV-ECMO initiation due to PaCO_2_ related reductions in cerebral blood flow and consequent hypoperfusion [14, 27]. Notwithstanding the limitations of the small sample size in our study, these biomarkers of neurologic injury did not appear to be related to changes in PaCO_2_. Given that 50 (87%) patients had COVID-19, it is important to emphasize that the interplay of VV-ECMO associated CNS injury with COVID-19 may be distinct from other respiratory pathology [28].

It should be noted that although NF-L and GFAP levels were greater in patients undergoing VV-ECMO with overt CNS injuries, their systemically circulating levels in patients without injury were greatly increased compared to normative values in healthy controls [27, 29]. This finding raises the possibility that there may be subclinical CNS injury following initiation of VV-ECMO that is not detectable on CT. The pathophysiologic pattern of injury, natural history, and clinical sequelae are unknown and represent key research areas for the future.

Our study should be viewed within the context of its strengths and also limitations. In terms of strengths, we conducted a prospective design with timed biomarker sampling in relation to the timing of VV-ECMO. Our study also used a highly sensitive analytical platform to assess brain biomarkers [30], which are closely related to clinical outcome assessment in patients with neurological injury, shown to have instantaneous release in setting of brain hypoxia and are brain-specific in their tissue of origin [27]. Limitations of this study include our relatively small sample size, single center study design and our selection of a composite outcome of intracerebral hemorrhage or ischemia to denote CNS injury. Importantly, these entities may represent different mechanisms of cerebral injury. Given the inability to do daily neurological examinations or CT imaging of patients on VV-ECMO, the timing of CNS injury remains unclear. Further, our number of CNS events is relatively small, and future work to assess the diagnostic utility of brain biomarkers in this population should be multicenter to increase statistical power and strengthen external validity and given our sample of convenience a sample size calculation was not performed. The number of CNS events also limits the ability to control for confounding in our study. Our study did not report descriptive outcomes of CNS injury such as modified Rankin scale, which would further enrich outcomes. Lastly, most patients in our cohort presented with COVID-19, which can result in delays in transportation for imaging and has been independently associated with neurological injury in patients who have not required VV-ECMO [29].

## Conclusions

Although rapid decreases in PaCO_2_ following initiation of VV-ECMO were greater in patients with CNS injuries versus those without, considerable data overlap and absence of obvious relationships with PaCO_2_ with brain biomarkers indicate that other pathophysiologic variables are at play. NF-L and GFAP were increased in critically ill patients undergoing VV-ECMO for acute respiratory failure in whom we had identified CNS injuries on head CT compared with those without.

### Supplementary Information

Below is the link to the electronic supplementary material.

Supplementary file1 (DOCX 587 KB)

Supplementary file2 (DOCX 18 KB)
