# Supplementary material for: Characterizing the Relationship Between Arterial Carbon Dioxide Trajectory and Serial Brain Biomarkers with Central Nervous System Injury During Veno-Venous Extracorporeal Membrane Oxygenation: A Prospective Cohort Study
Source: Neurocrit Care. 2024 Feb 1;41(1):20–8. doi: 10.1007/s12028-023-01923-x (PMC11335840; doi:10.1007/s12028-023-01923-x)
Supplement: Supplementary file 1 — Supplementary file1 (DOCX 587 KB) [file 12028_2023_1923_MOESM1_ESM.docx]

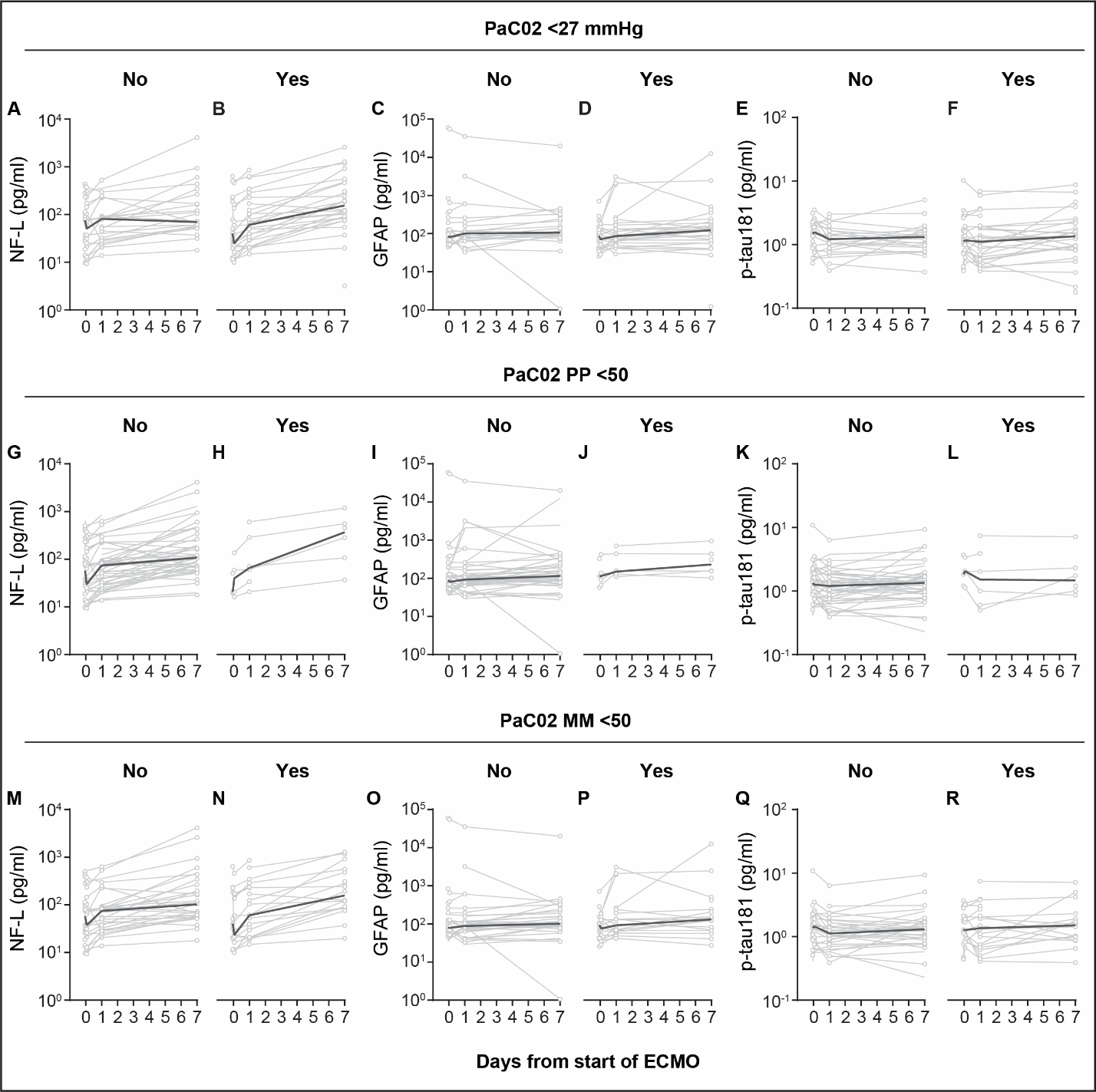


**E-Figure 1**. **Neurological biomarker trajectories stratified by arterial carbon dioxide thresholds on VV-ECMO.** Connected line plots of neurologic biomarker concentration (y-axis) at four time points (x-axis): pre-VV-ECMO, and 1-hour, 24-hours and 7-days post-VV ECMO stratified by ∆PaCO_2_ thresholds (≥27 mmHg vs. <27 mmHg; PP ≥50% vs. < 50%; MM ≥50% vs. <50%). Each light grey line is an individual patient. The black line is a locally weighted scatterplot smoothing function across all patients.
