# Supplementary material for: Characterizing the Relationship Between Arterial Carbon Dioxide Trajectory and Serial Brain Biomarkers with Central Nervous System Injury During Veno-Venous Extracorporeal Membrane Oxygenation: A Prospective Cohort Study
Source: Neurocrit Care. 2024 Feb 1;41(1):20–8. doi: 10.1007/s12028-023-01923-x (PMC11335840; doi:10.1007/s12028-023-01923-x)
Supplement: Supplementary file 2 — Supplementary file2 (DOCX 18 KB) [file 12028_2023_1923_MOESM2_ESM.docx]

**E Table 1**. Association of arterial carbon dioxide and detection of CNS injury in patients on VV-ECMO .

|  | No CNS injury (n=47) | CNS Injury (n= 12) | Odds Ratio | 95% CI | p value |
| --- | --- | --- | --- | --- | --- |
| PaCO_2_ pre VV-ECMO, mmHg [IQR] | 67 [54-76] | 71 [63-96] | 1.0 | 0.99, 1.03 | 0.94 |
| PaCO_2_ reduction on initiation of VV-ECMO >27mmHg | 22/47 (47) | 8/12 (67) | 1.0 | 0.98, 1.03 | 0.29 |
| PaCO_2_ max first 24 hours, mmHg [IQR] | 69 [60-89] | 96 [66-107] | 1.0 | 0.99, 1.03 | 0.24 |
| PaCO_2_ _max-min_, mmHg [IQR] | 28 [19-44] | 43 [33-67] | 1.0 | 0.99, 1.05 | 0.09 |
| ∆PaCO_2_ 50%_pre-post%_* | 4/47 (8.5) | 3/12 (25) | 3.6 | 0.68, 18.85 | 0.13 |
| ∆PaCO_2 >_ 65%_max-min%_ † | 8/47 (17) | 5/12 (42) | 3.5 | 0.88, 13.80 | 0.08 |
| ∆PaCO_2 >_ 60%_max-min%_ † | 10/47 (21) | 7/12 (58) | 5.2 | 1.4, 19.9 | 0.02 |
| ∆PaCO_2 >_ 55%_max-min%_ † | 12/47 (26) | 8/12 (67) | 5.8 | 1.45, 22.90 | 0.01 |
| ∆PaCO_2 >_ 50%_max-min%_ † | 12/47 (26) | 9/12 (75) | 8.8 | 2.02, 37.75 | <0.01 |
| ∆PaCO_2 >_ 45%_max-min%_ † | 18/47 (38) | 9/12 (75) | 4.8 | 1.15, 20.26 | 0.03 |
| ∆PaCO_2 >_ 40%_max-min%_ † | 22/47 (47) | 10/12 (83) | 5.7 | 1.12, 28.79 | 0.04 |
| ∆PaCO_2 >_ 35%_max-min%_ † | 26/47 (55) | 10/12 (83) | 4.0 | 0.79, 20.47 | 0.09 |
| ∆PaCO_2 >_ 50%_max-min%_ †  Adjusted for COVID-19 |  |  | 8.8 | 2.03, 38.10 | <0.01 |

_*(_24-h post-ECMO PaCO_2_ - pre-ECMO PaCO_2_)/pre-ECMO PaCO_2_

† (PaCO_2_ max first 24 hours- PaCO_2_ min first 24 hours)/Pre ECMO PaCO_2_

*ECMO* extracorporeal membrane oxygenation

**E-Table 2. Comparison of plasma NF-L, GFAP, and ptau181 taken at 4 timepoints over the course of VV-ECMO (prior to and 1h, 1 day, and 7 days post) in patients stratified by the presence of absence of subsequent CNS injury.**

| Biomarker (pg/ml) |  | Pre-VV-ECMO | 1h Post-VV-ECMO | Day 1 | Day 7 |
| --- | --- | --- | --- | --- | --- |
| NFL median [IQR] number | No-injury  n=47 | 40.1  [19.8-87.6] n=29 | 27.5  [13.1-70.9] n=26 | 55.9  [28.6-88.3] n=42 | 102.7  [59.3-197] n=42 |
|  | CNS injury  n=12 | 312.1  [19.6-435] n=6 | 184  [16.2-375] n=6 | 293  [92.7-530]  n=10 | 456 [192-11180] n=10 |
|  | P value | 0.02 | 0.02 | 0.01 | 0.06 |
| GFAP median [IQR]  number | No- injury | 73.7  [56.1-103]  n=29 | 74.3  [49.9-116] n=29 | 85.8  [64.7-117] n=42 | 103  [74.4-144] n=42 |
|  | CNS injury | 153  [26.5-238] n=6 | 156  [51.2-189] n=6 | 385 [104-2092] n=10 | 29  [97.6-2456] n=10 |
|  | P value | 0.11 | 0.91 | 0.10 | 0.02 |
| p-tau 181 median [IQR]  number | No-injury | 1.21  [0.79-2.03] n=29 | 1.24  [0.82- 2.00] n=29 | 1.01  [0.73-1.84] n=42 | 1.34  [0.78- 1.69] n=42 |
|  | CNS injury | 1.88  [1.34-2.8] n=6 | 1.66  [1.45-2.07] n=6 | 1.56  [1.11-3.04] n=10 | 1.22  [1.02-1.9] n=10 |
|  | P value | 0.72 | 0.31 | 0.08 | 0.42 |
